# Study on the Attribute Characteristics of Road Cracks Detected by Ground-Penetrating Radar

**DOI:** 10.3390/s25030595

**Published:** 2025-01-21

**Authors:** Shili Guo, Mingyu Yu, Zhiwei Xu, Guanghua Yue, Wencai Cai, Pengfei Tian

**Affiliations:** 1 Institute of Environmental and Biological Engineering, Henan University of Engineering, Zhengzhou 451191, China; guoshili@haue.edu.cn (S.G.); ymy_2016@126.com (M.Y.); 2 School of Geophysics and Geomatics, China University of Geosciences, Wuhan 430074, China; 3 Key Laboratory of Road and Traffic Engineering of the Ministry of Education, Tongji University, Shanghai 201804, China; 24310366@tongji.edu.cn; 4 Henan Wanli Transportation Science and Technology Group Nondestructive Testing and Reinforcement Technology Co., Ltd., Xuchang 461001, China; hywc2018@163.com (W.C.); tiansleep@163.com (P.T.)

**Keywords:** ground-penetrating radar (GPR), road cracks, attribute characteristics, early-time signals (ETSs), amplitude change rate

## Abstract

Cracks are a common form of road distress that can significantly impact pavement integrity. Accurate detection of the attribute characteristics of cracks, including the type, location (top and bottom), width, and orientation, is crucial for effective repair and treatment. This study combines numerical simulations with filed data to investigate how the amplitudes of ground-penetrating radar (GPR) early-time signals (ETSs) vary with changes in the crack top and width, as well as how variations in the crack bottom impact radar reflected wave amplitude. The results show that when GPR ETSs are mixed with diffracted waves from the crack top, the amplitude change percentage of the ETS at the crack top exhibits a pronounced ‘∨’-shaped dip, which provides a clearer indication of the crack top. Furthermore, a positive correlation exists between crack width and the amplitude change percentage, offering a theoretical basis for quantitatively estimating crack width. On the reflected wave originating from the interface between the semi-rigid base and the subgrade, a pronounced ‘∧’-shaped dip is observed in the trough amplitude change percentage of the reflected wave at the crack bottom. For cracks of the same width, the amplitude of the ‘∧’ vertex from reflective cracks is approximately three times greater than that from fatigue cracks. This discrepancy helps identify the crack bottom and quantitatively diagnose their types. The line connecting the vertices of the ‘∨’ and ‘∧’ shapes indicate the crack’s orientation. Accurate diagnosis of crack properties can guide precise, minimally invasive treatment methods, effectively repairing road cracks and extending the road’s service life.

## 1. Introduction

By the end of 2023, the total length of highways in China had exceeded 183,600 km. Following the “strong foundation, thin surface” design concept, these highways are typically constructed with a pavement structure consisting of a semi-rigid base and an asphalt surface layer [1]. Reflective and fatigue cracks are the two primary types of road cracks that are commonly observed within China’s highways. Under the repeated effects of temperature, humidity, and vehicle loads, the semi-rigid base will contract and crack, progressively extending upward into the asphalt surface, thus forming reflective cracks [2]. Once these reflective cracks penetrate both the semi-rigid base and the asphalt surface, various types of water can then seep into the road structure along the cracks. This infiltration leads to asphalt aging, accelerated deterioration of the surface and base layers, and significantly impacts overall durability [3,4,5]. This also can lead to more severe damage in the deep layers of the road, including water-saturated subgrade, soil loosening, differential settlement, cavities, and voids [6]. Fatigue cracks are primarily caused by repetitive loading and fatigue stress from heavy vehicles [7], and they typically appear on the asphalt surface. In contrast, reflective cracks extend through both the semi-rigid base and the asphalt layer. Given that cracks can form at different layers of the pavement, it is crucial to accurately detect their characteristics for appropriate treatment strategies.

Road crack detection methods can be categorized into two major types: fixed-point and mobile measurement methods. Fixed-point measurement methods include core drilling, distributed fiber optic sensing, and ultrasonic crack detection. Core drilling can damage the road surface. Distributed fiber optic sensors, equipped with protective sleeves, can not only detect the onset of cracks but also accurately measure their opening widths [8]. However, the sensors must be preinstalled at critical locations within the road structure. Ultrasonic detection technology assesses the presence, location, size, and type of cracks by analyzing the parameters of reflected signals [9]. In practice, the defects common to the aforementioned fixed-point methods for crack detection are inefficient and time-consuming. Mobile measurement methods involve using vehicles or drones equipped with lasers [10], infrared thermography [11], remote sensing [12], cameras [13], and other devices to capture images of road structures that may contain cracks. These images are then processed using traditional image processing techniques or artificial intelligence algorithms to segment, identify, and extract geometric parameters of the cracks. This method is highly efficient and can accurately quantify the geometric attributes and distribution characteristics of cracks on the road surface. However, the detection depth of mobile measurement methods is limited, making it difficult to probe the entire morphology of cracks. Especially since cracks extending to the surface are typically treated with slotting and asphalt sealing to prevent surface water infiltration, effectively filling and covering the crack tops with asphalt, the aforementioned mobile detection technologies cannot accurately determine crack locations, estimate crack widths, or understand the internal development of cracks.

Ground-penetrating radar (GPR) is a geophysical method that utilizes high-frequency radio waves (ranging from 10^6^ to 10^9^ Hz) to detect electrical anomalies within subsurface medium [14]. It offers several advantages, including high precision, efficiency, continuous non-destructive testing, real-time imaging, and intuitive results [15,16,17], making it a primary technology for the long-distance, engineered, and efficient non-destructive detection of road cracks [18,19]. Ground-coupled shielded antennas, recognized for their strong resistance to electromagnetic interference, environmental adaptability, broad frequency range, deep penetration, and high spatial resolution, are highly sensitive to subtle electrical differences in structures and are widely utilized for detailed road crack detection [17]. To maximize electromagnetic wave penetration into the underground medium, ground-coupled shielded antennas must be positioned as close to the surface as possible. In this configuration, the air and ground waves, with minimal offset, cannot be separated and instead overlap, resulting in the GPR early-time signal (ETS) [20,21]. The amplitude, waveform, and phase of this early-time signal (ETS) are closely related to the electrical parameters of the shallow subsurface medium within its detection depth range [22,23]. In homogeneous media, the top and bottom of vertical cracks correspond to separate diffracted waves with opposite phases, facilitating the easy identification and localization of their top and bottom [18,24]. However, if a crack extends to the road surface, its diffracted wave mixes with the ETS, masking the crack top and significantly interfering with its detection. Additionally, lower-frequency antennas result in longer ETS durations, a greater depth of influence, and increased interference, complicating the identification and localization of crack top [25].

Normally, road structure is typically a layered medium. GPR profiles of the layered medium with a vertical crack reveal that diffracted waves can not only be generated at its top and bottom [26] but also at each interface. The diffracted wave occurring at each interface might be additional multiple diffracted waves. These additional waves resemble the diffracted waves from the crack bottom in both position and waveform, which may potentially lead to errors in accurately determining the location of the crack bottom [27]. Since it is not easy to accurately locate the crack top and bottom, it is also impossible to determine the crack orientation. The lateral resolution of GPR is determined by the radius of the first Fresnel zone. When the crack width exceeds this radius, diffracted waves from both edges of the crack top become clearly visible on the GPR profile, enabling direct measurement of the crack width. However, the road crack width is typically much smaller than the first Fresnel zone radius. Directly measuring the crack width from GPR profiles is still challenging. Despite this, the amplitude of the diffracted wave at the crack top increases significantly with crack width, providing a qualitative basis for comparing relative widths. However, without calibration values, a quantitative estimation of crack width is not feasible [28,29]. Simulated GPR data of reflective and fatigue cracks suggest that the amplitude differences of the reflected wave generated at the interface between the semi-rigid base and subgrade caused by reflective cracks are significantly greater than those caused by fatigue cracks. However, this amplitude difference has yet to be accurately quantified [30].

The GPR method faces challenges in accurately locating the top and bottom of cracks, diagnosing crack orientation, calculating crack widths, and quantitatively determining crack types. These limitations hinder the effective implementation of targeted “minimally invasive” repair measures for road cracks. When the crack top is near the road surface, the diffracted wave will mix with the ETS, leading to localized changes in the amplitude of the ETS. To investigate this, this study employs numerical simulations to examine how the amplitude of the ETS varies with the width of the surface crack. The first trough amplitude of the ETS can serve as a baseline to quantify the relationship between crack width and the percentage change. This approach enables accurate crack top localization and crack width estimation. By referencing the trough amplitude of reflected waves at the interface between the semi-rigid base and the subgrade as a reference, the study quantifies amplitude percentage changes by fatigue and reflective cracks of varying widths. This improves crack bottom localization, crack type diagnosis, and crack orientation assessment. Based on the crack properties detected with GPR, targeted, minimally invasive crack repair measures can be conducted on the highways.

## 2. Methodology

### 2.1. Air and Ground Waves

**Figure 1:**
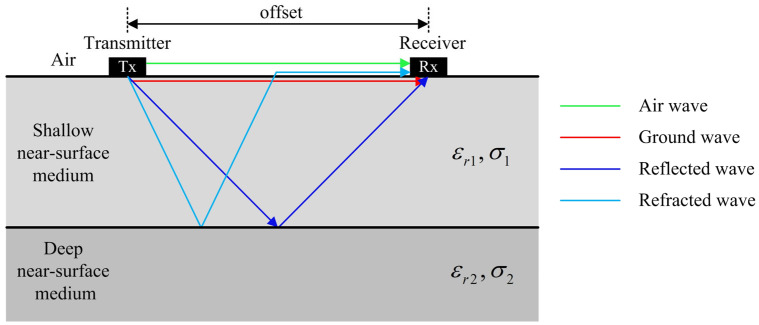
Schematic diagram of electromagnetic wave propagation between the transmitter and receiver of a ground-coupled GPR system in the shallow subsurface layer. $\varepsilon_{r1}$ and $\varepsilon_{r2}$ are the relative permittivity of the near-surface shallow and deeper medium, respectively, $\sigma_{1}$ and $\sigma_{2}$ are the corresponding electrical conductivities. For a better display of the ETS, the thicknesses of the two media are not in scale.

Figure 1 illustrates the electromagnetic wave propagation between the transmitter and receiver placed on the ground surface. The airwave refers to the electromagnetic wave that travels directly from the transmitter to the receiver through the air (green arrow). The ground wave, on the other hand, travels through the very shallow near-surface medium (red arrow). Compared to the reflected (blue arrow) and refracted waves (cyan arrow), the air and ground waves have shorter propagation distances, less energy loss, and stronger amplitudes. The amplitudes of the air and ground waves can be expressed as follows:

(1) $$A_{air-wave}=\frac{\sqrt{\varepsilon_{0}\mu_{0}}}{2\pi \varepsilon_{0}\left(\varepsilon_{r1}-1\right)S^{2}},$$

(2) $$A_{ground-wave}=-\frac{\sqrt{\varepsilon_{0}\varepsilon_{r1}\mu_{0}}}{2\pi \varepsilon_{0}\left(\varepsilon_{r1}-1\right)S^{2}}\cdot \exp\left[-\left(\frac{1}{2}\right)\frac{\sqrt{\mu_{0}}}{\varepsilon_{0}\varepsilon_{r1}}\sigma_{1}S\right],$$

where $\varepsilon_{0}$ represents the permittivity of vacuum; $\varepsilon_{r1}$ and $\sigma_{1}$ represent the relative permittivity and electrical conductivity of the near-surface shallow medium, respectively; and $\mu_{0}$ denotes the permeability of vacuum. S is the offset, i.e., the distance between transmitter and receiver antennas. The exponential decay term represents the attenuation characteristics of the ground wave, and the negative sign indicates that the ground wave has the opposite polarity to the airwave. As illustrated in Equations (1) and (2), the amplitudes of both air and ground waves are inversely proportional to the relative permittivity of the near-surface shallow medium [31].

Furthermore, the amplitude of a ground wave is influenced by the electromagnetic parameters of the medium within its detection depth. The detection depth of the ground wave is related to the antenna’s central frequency, offset, and the permittivity of the shallow near-surface medium. It is inversely proportional to the antenna’s main frequency; a lower frequency results in a greater detection depth. It is directly proportional to the offset distance. To be specific, a larger offset distance leads to a greater detection depth. A higher relative permittivity of the near-surface medium results in a shallower detection depth.

### 2.2. The Amplitude and Detection Depth of ETS

Multi-offset measurements like common midpoint (CMP) or wide-angle reflection and refraction (WARR) are used to measure the airwave and ground wave. As the offset between the transmitter and receiver increases, the air and ground waves gradually separate on the time–domain profile due to their different propagation speeds. However, multi-offset measurements are only suitable for systems with separate transmitter and receiver antennas [23]. Particularly, commercial GPR systems that utilize ground-coupled shielded antennas typically feature an integrated transmitter-receiver design. These antennas have fixed positions for the transmitter and receiver, making multi-offset measurements impractical. For example, the SIR and LTD series of ground-coupled shielded antennas operate at central frequencies of 1500 MHz, 900 MHz, 400 MHz, and 270 MHz, with fixed transmitter-receiver offsets of 6 cm, 15 cm, 16 cm, and 24 cm, respectively. Consequently, these systems can only employ fixed-offset profiling methods. Due to the small offset between the antennas, the air and ground waves cannot be distinguished individually. Instead, they couple and superimpose to form the ETS.

The detection depth of ETSs is primarily determined by the ground wave [22]. In conventional profile measurements, since the offset distance between antennas is fixed and the antenna’s main frequency and wave speed are functions of the wavelength, the detection depth of the ground wave is typically expressed as a function of wavelength. For instance, the detection depth of ground waves is about 0.17 to 0.6 times the operating wavelength of the GPR system in a given material [32,33]. For a 900 MHz antenna with a wavelength of 11.11 cm in concrete with a relative permittivity of 9, the detection depth ranges from 1.9 cm to 6.7 cm.

**Figure 2:**
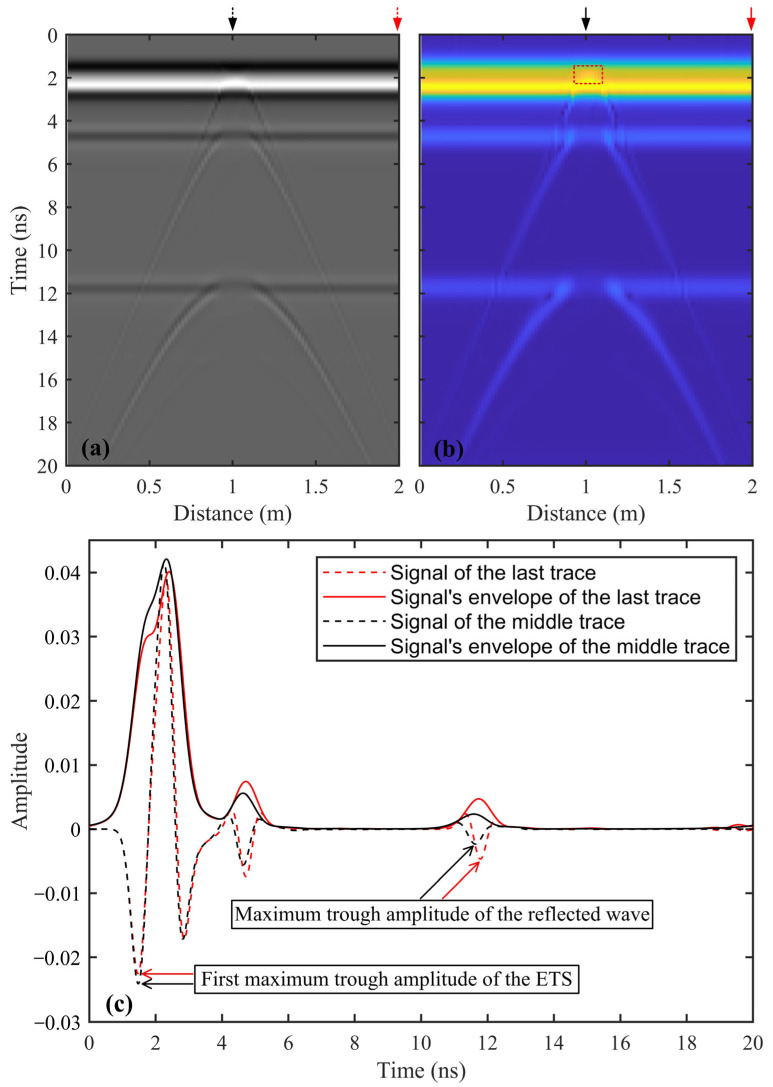
(**a**) Simulated GPR profile of the highway model with a vertical crack situated at the road surface, and (**b**) is the envelope of the simulated GPR profile shown in (**a**). The details about the highway model and numerical simulation can be found in Section 3.1. The arrows denote the locations of the two selected traces of the GPR profile and corresponding envelope, and the red dashed box indicates the amplitude enhancement caused by the top of the crack. (**c**) Waveforms of two selected GPR traces and their corresponding envelopes versus Time.

In a GPR profile, an ETS usually exhibits strong amplitudes, stable waveforms, and clear phases, with a duration of approximately equal to one signal period. When the top of a crack lies within the detection depth of the ETS, the diffracted wave generated by the crack top may superimpose with the ETS (Figure 2a). This superposition can significantly obscure the diffracted waves from the crack top, making the identification and localization of the crack top more challenging. However, the amplitude of the ETS, which results from the superposition of the airwave and the ground wave, also exhibits a clear inverse proportionality to the permittivity of the near-surface shallow medium. Therefore, when the cracks are filled with air, the amplitude intensity of the corresponding ETS is significantly enhanced compared to the surrounding medium. In contrast, when the crack is filled with water, the amplitude intensity of the corresponding ETS is notably reduced. This characteristic provides a basis for identifying the crack top.

### 2.3. Analysis of ETS Amplitude

The average envelope amplitude (AEA) statistic, introduced by Pettinelli et al. [21], is used to analyze ETSs. Unlike traditional methods that focus on the velocity of ground or reflected waves, the AEA statistic examines variations in the amplitude of ETSs. This provides valuable insights into the dielectric properties of the shallow subsurface. The Hilbert transform is employed to calculate the AEA of the ETS, and it is described by the following equation:

(3) $$\hat{x}(t)=\frac{1}{\pi }\int_{-\infty }^{\infty }\frac{x(s)}{t-s}ds,$$

where $\hat{x}(t)$ is the Hilbert transform of x(t), and the integral is the Cauchy principle value integral [31]. By calculating the absolute value of the transformed GPR trace, the Hilbert transform enables the determination of the envelope of the trace, which is also known as the instantaneous amplitude. When the top of a crack is located at the road surface, the dielectric constant of the air within the crack is significantly lower than that of the surrounding asphalt concrete. Because the amplitude of the ETS is inversely proportional to the dielectric constant of the shallow subsurface, the average envelope amplitude corresponding to the crack top is noticeably enhanced (indicated by the red dashed box in Figure 2b). This localized increase in amplitude indicates the position of the crack top. Although this method can identify the crack top, it is inefficient and unsuitable for processing long-range GPR profiles containing hundreds of cracks.

Thus, this paper proposes the use of an extreme value method to track the first maximum trough amplitude within the ETS (as shown in Figure 2b) and to statistically analyze the amplitude variation curve. This method holds promise as a faster and more intuitive way to evaluate long-range GPR data containing hundreds of cracks.

## 3. Numerical Tests and Results

### 3.1. Attribute Parameters of Vertical Cracks

**Figure 3:**
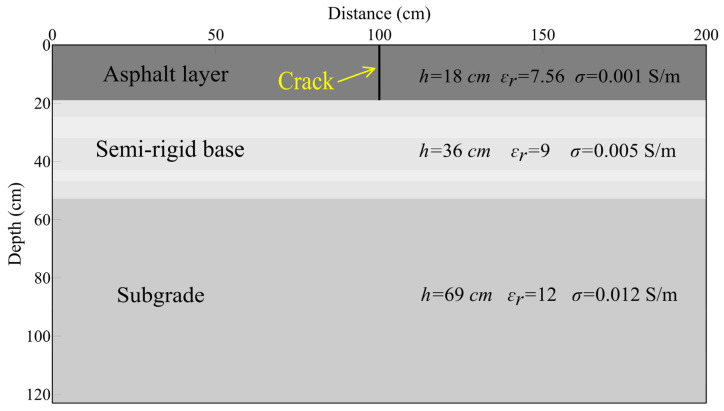
Schematic diagram of the highway model with a vertical fatigue crack. Note that the crack width is not to scale here.

The highway structure layer is a typical layered medium, consisting of an asphalt surface layer, a semi-rigid base layer, and a fill subgrade from top to bottom. For simplicity, the highway structure is simplified as a three-layered uniform medium (Figure 3). Typically, the thicknesses of the asphalt surface layer, semi-rigid base layer, and subgrade within most of the in-service highways in China are approximately 18 cm, 36 cm, and 69 cm, respectively. To simulate fatigue cracks in such highway structures, we established a highway model where the crack extends vertically through the entire asphalt surface layer, which is indicated by the yellow arrow in Figure 3. The model has a spatial dimension of 1075 × 615 grids (width × height) with a spatial step size of 0.2 cm. The model is surrounded by an absorbing layer with a thickness of 10 grids. The relative permittivities of the asphalt surface layer, semi-rigid base layer, and subgrade are 7.56, 9, and 11, respectively. The electrical conductivities of the three layers are 0.001 S/m, 0.005 S/m, and 0.012 S/m, respectively. The dielectric parameters of the three layers in this study are derived from core sampling verification results and accumulated experience from GPR detection of road structure layers. Numerous studies suggest that the width of road cracks typically ranges from 0 to approximately 2 cm. It is important to note that, to systematically investigate how the radar wave field response varies with crack width, we established 11 different crack widths, ranging from 0.2 cm to 2.2 cm in increments of 0.2 cm. All of the 11 cracks are filled with air, which has a permittivity of 1 and electrical conductivity of 0 S/m. Due to space limitations, only a schematic diagram of the 11 highway models is presented here.

**Figure 4:**
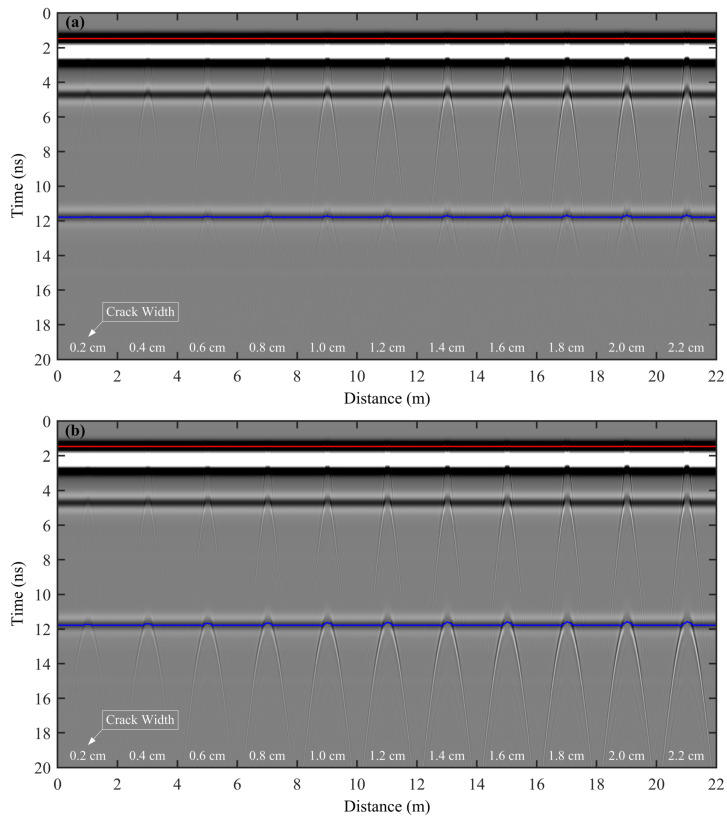
Simulated GPR profiles of 11 highway models with a varying width of fatigue crack (**a**) and reflective crack (**b**). White text shows the different crack widths of the crack within the highway model shown in Figure 3. Red and blue curves denote the first maximum trough amplitude of ETS and the maximum trough amplitude of the reflected wave generated at the interface between the semi-rigid base layer and subgrade.

The finite difference time domain (FDTD) method was used for radar forward modeling, and the convolutional perfectly matched layer (CPML) absorbing boundary conditions method was used to absorb the outgoing waves [34]. The total recording time was 20 ns, with a time sampling interval of 0.0047 ns. The excitation pulse used was a normalized Ricker wavelet with a central frequency of 900 MHz. Additionally, the transmitter and receiver antennas were positioned 0.2 cm above the asphalt surface layer, with a fixed offset of 15 cm. The two antennas were moved synchronously from left to right with a spacing of 2 cm, resulting in a total number of 100 radar records for each model. In total, we obtained 1100 radar records for the 11 established highway models with different fatigue cracks. For a better comparative analysis of the relationship between ETS amplitude and crack width, the simulated GPR profiles of the 11 highway models were concatenated in the increasing crack width, resulting in a single GPR profile (Figure 4a). By fixing the position of the crack top, as shown in Figure 3, and extending the crack bottom to the base of the semi-rigid layer, the fatigue crack can be converted into a reflective crack. We then conducted the GPR simulation by keeping other model parameters and simulation parameters unchanged. The joint simulated GPR numerical simulation profile for 11 highway models with a varying width of reflective crack is shown in Figure 4b.

In Figure 4, two clear reflected events, which correspond to the reflected waves generated at the two internal interfaces of the highway model, can be observed at approximately 4.6 ns and 11.8 ns. The amplitude of the ETS is much higher than that of the reflected and diffracted waves. With the increase in crack width, the diffraction hyperbolas of the crack top and bottom become more and more obvious. An interesting phenomenon is that the diffraction hyperbolas can be found at about 11.8 ns, and they are superposed with the nearby reflected events. In Figure 4, the polarity sequences of the reflected waves at the boundaries between the asphalt surface layer and the semi-rigid base layer, as well as between the semi-rigid base layer and the subgrade, exhibit a “positive-negative-positive” pattern. This is contrary to the polarity sequence observed in the ETS, indicating that the reflection coefficients at these boundaries are negative. Additionally, Figure 4 shows that the strong amplitude, stable waveform, and clear phase of the ETS—lasting approximately one signal period—obscure the diffracted wave information related to the crack top. As a result, it is difficult to identify and differentiate the diffracted wave image and the location of the crack top in the GPR profile, significantly complicating the task of pinpointing its exact position.

As can be observed from Figure 4, it is evident that the fatigue crack models, aside from displaying the diffracted waves associated with the top and bottom of reflective cracks, also exhibit an additional multiple diffracted waves at the interface between the semi-rigid base and the subgrade. These multiple diffracted waves align with the phase axis of the boundary-reflected wave and share a comparable position and shape with the diffracted wave observed at the bottom of the reflective crack. Such similarity can potentially result in the misinterpretation of the fatigue crack bottom.

**Figure 5:**
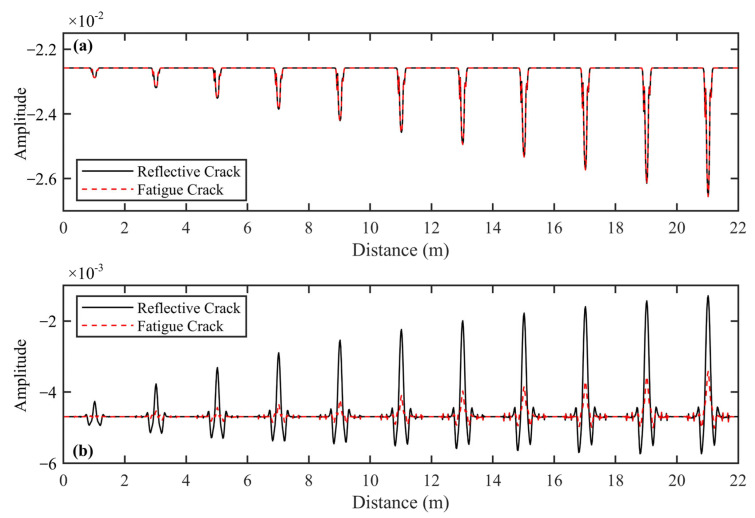
Amplitude variation curves with varying crack width for 11 highway models with a varying width of fatigue/reflective crack highway. (**a**) The first maximum trough amplitude of ETS. (**b**) The maximum trough amplitude of the reflected wave is generated at the interface between the semi-rigid base layer and subgrade.

The lateral resolution of a GPR system is determined by the Fresnel zone radius. If the crack width exceeds this radius, the GPR profile will clearly display diffracted waves from both edges of the crack, enabling direct identification of the crack edges and measurement of its width. However, in Figure 4 and Figure 5, the maximum crack width is only 2.2 cm, which is significantly smaller than the GPR’s lateral resolution. As a result, the shape and size of the diffracted waves at the top and bottom of the crack remain unchanged as the crack width increases. Instead, their amplitude increases with the crack width, exhibiting a clear and predictable pattern.

**Figure 6:**
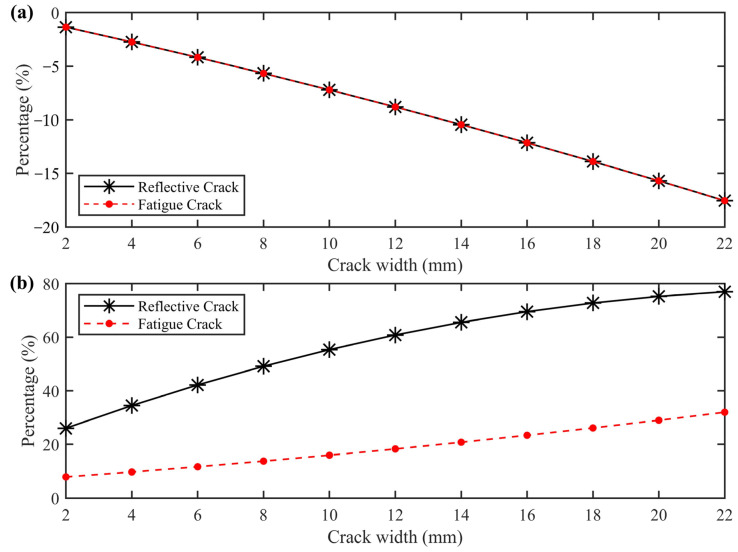
Fitted curves of amplitude variation percentages with crack width for fatigue and reflective cracks. (**a**) The fit-ted curve of the percentage change in amplitude with crack width for the first maximum trough amplitude of ETS. (**b**) Fitted curve of the percentage change in amplitude with crack width for the maximum trough amplitude of the reflected wave at the interface between the semi-rigid base and the subgrade.

Based on the method described in Section 2, we extracted the first maximum trough amplitude of the ETS (red curves in Figure 4) and the maximum trough amplitude of the reflected wave generated at the interface between the semi-rigid base layer and subgrade (blue curves in Figure 4). To study the relationship between crack properties (including width and type) and amplitude intensity, we extracted the amplitude values whose positions were marked by the two curves with different colors in Figure 5 and Figure 6. To minimize interference from other reflected signals that could affect the signal-to-noise ratio of the ETS, we focused our analysis on the first maximum trough amplitude of the ETS.

Figure 5a shows the first maximum trough amplitude variation curves of the ETS for fatigue and reflective cracks. The amplitude intensity of the ETS changes significantly at the crack locations due to diffracted waves from the tops of these cracks. The amplitude curves form a ‘∨’ shape, with the vertex indicating the crack top. Importantly, the two amplitude curves overlap completely, suggesting that the bottoms of both crack types are beyond the effective detection depth of the ETS. Therefore, the amplitude intensity at the ‘∨’ vertex is only affected by the position and width of the crack top, not the crack bottom. Furthermore, as the crack widens, there is a clear increase in amplitude intensity at the ‘∨’ vertex. Figure 5b displays the maximum trough amplitude variation curves of reflected waves at the interface between the semi-rigid base and the subgrade. The amplitude of the reflected wave at the crack bottom undergoes intense changes due to the influence of the diffracted wave at the bottom of the reflective crack or multiple diffracted waves at the bottom of the fatigue crack. The amplitude variation curve features a noticeable protrusion shaped like a ‘∧’, indicating a significant increase. As crack width increases, a notable increase in the amplitude of the reflected wave is observed for both crack types, indicating a clear pattern. However, when comparing cracks of the same width, the amplitude of the reflected wave at the ‘∧’ vertex is significantly higher for reflective cracks than for fatigue cracks at the interface.

To quantify the variation of amplitude values at the ‘∨’ vertex with crack width, we used the mode of the amplitude values at the first trough of the ETS from Figure 5a as a reference. The amplitude value is not affected by the diffracted wave of the cracks. We then analyzed the rate of change in amplitude values for different crack widths. The expression for the percentage change rate *R* of amplitude values under different crack widths is as follows:

(4) $$R=\frac{Mode-A_{crack}}{Mode}\times 100\%,$$

where ‘Mode’ is the value that appears most frequently among the first maximum trough amplitude in the ETS or the maximum trough amplitude of the reflected wave from the semi-rigid base and the subgrade. $A_{crack}$ is the amplitude value of the crack top or bottom at the first maximum trough amplitude in the ETS or the maximum trough amplitude of the reflected wave from the semi-rigid base and the subgrade.

We used the least squares method to perform a polynomial fit and established the relationship between crack width and the amplitude change rate. The fitting results are shown in Figure 6a, where the *x*-axis represents crack width, and the *y*-axis represents the percentage change in amplitude values. The functional relationship is given by the following:

(5) $$y=-0.0064x^{2}-0.6554x-0.0092,$$

From Equation (5), it can be seen that the relationship between crack width and the corresponding change rate of amplitude values is approximately linear. This provides a theoretical basis for quantitatively estimating crack width.

Using the mode of the reflected wave amplitude at the interface as a reference, Figure 6b illustrates the percentage change in amplitude for both reflective and fatigue cracks as crack width increases. When comparing cracks of the same width, the percentage change in amplitude for reflective cracks is approximately three times greater than that for fatigue cracks. Notably, for a reflective crack with a width of 2 mm and a fatigue crack with a width of 18 mm, the percentage change in amplitude at the interface is comparable. This finding offers a theoretical basis for determining the location of the crack bottom and distinguishing between the two types of cracks.

### 3.2. Attribute Parameters of Inclined Cracks

**Figure 7:**
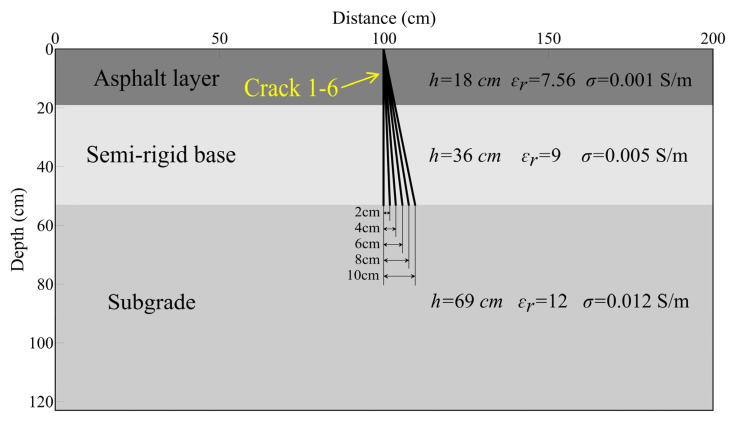
Schematic diagram of the highway models which contains reflective cracks with different orientations. From left to right, the six cracks, numbered 2 to 6, correspond to the six different highway models.

**Figure 8:**
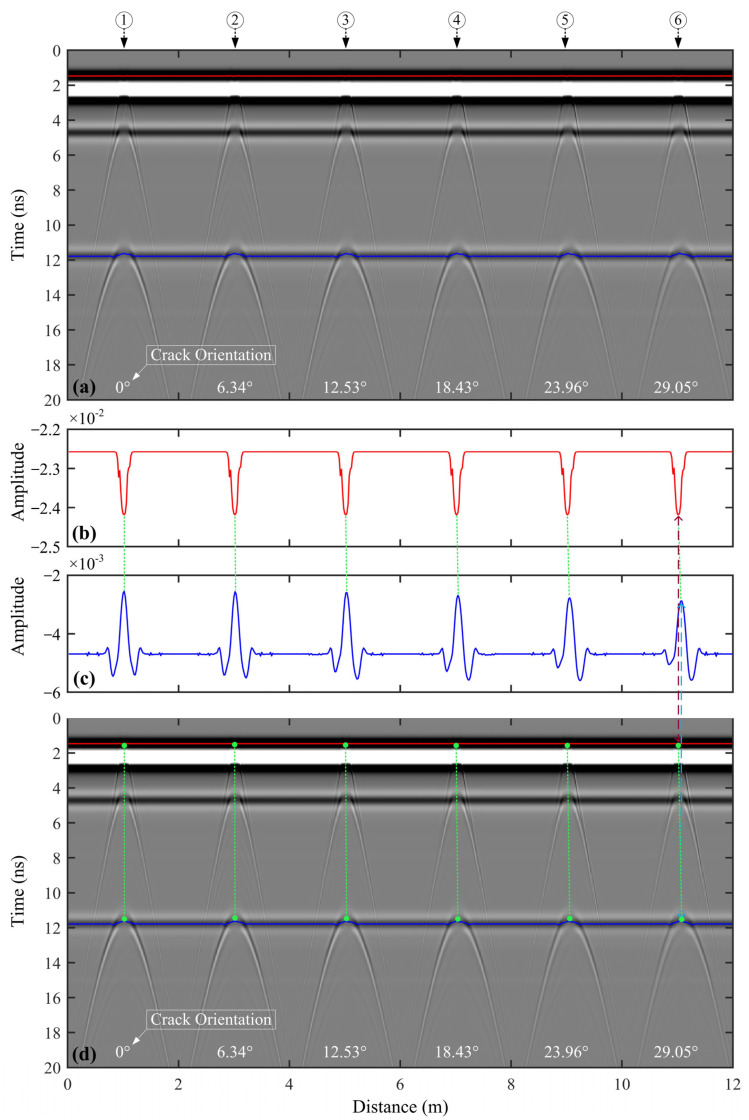
(**a**) Simulated GPR profiles of the 6 highway models shown in Figure 7. (**b**) The first maximum trough amplitude of the ETS. (**c**) The maximum trough amplitude of the reflected wave generated by the interface between the semi-rigid base and the subgrade. (**d**) Simulated GPR profiles of the 6 highway models are shown in Figure 7, with the locations of the crack top and bottom plotted as green dots. In panel (**a**,**b**), the red and blue curves denote the first maximum trough amplitude of ETS and the maximum trough amplitude of the reflected wave generated by the interface between the semi-rigid base layer and subgrade. The brown arrows point to the vertex of the “∨” and the crack top, while the cyan arrows point to the vertex of the “∧” and the crack bottom.

Vertical cracks are commonly found in road structural layers, though inclined cracks also occur. To better understand the GPR wavefield response of inclined cracks, we designed six highway models, each of which contains a reflective crack with a distinctive inclination angle, to investigate the relationship between the top width of inclined cracks and the amplitude of the ETS. For saving space limitations, Figure 7 is the schematic diagram of the six highway models because the only difference between them is the inclination angle of the cracks within them. Thus, we plot six different cracks in the three-layered highway model to represent all six models. The thicknesses and dielectric parameters of the three layers of the six models are the same as the models used in Section 3.1. Crack 1 is a vertical reflective crack, while the other five cracks are inclined reflective cracks. The locations of the crack top and the width of the six cracks are all the same, but the locations of the bottom of cracks 2 to 6 are progressive with an offset of 2 cm to the right side of crack 1 (Figure 7). The corresponding inclination angles between the five inclined cracks and the vertical direction are 6.34°, 12.53°, 18.43°, 23.96°, and 29.05°, respectively. These inclination angles are basically within the range of inclination angles found in actual inclined cracks. We performed forward modeling of the GPR wavefield for these six cracks, using a normalized Ricker wavelet with a dominant frequency of 900 MHz as the excitation source. For a better comparison and statistical analysis, we also concatenated the six numerical simulation profiles of the six cracks, as shown in Figure 8.

In Figure 8a, the diffracted wave at the top of the vertical crack ① shows bilateral symmetry, with energy evenly distributed on both sides. In contrast, the rightward-inclined cracks (② to ⑥) exhibit a higher energy level on the right side of their diffracted wave compared to the left. Furthermore, as the inclination angle of these cracks increases, the energy disparity between the two sides becomes more pronounced.

In Figure 8b, the first maximum trough amplitude of the ETS is the same for both the vertical crack ① and the inclined cracks (② to ⑥). This indicates that the first maximum trough amplitude in the ETS is solely dependent on the width of the crack top and is not influenced by the crack orientation. Compared to vertical crack, the tops and bottoms of inclined cracks not only have ‘∨’ and ‘∧’ shapes, respectively, but also the line connecting the ‘∨’ and ‘∧’ aligning with the crack orientation, as shown in Figure 8c. This distinctive feature provides a clearer, more intuitive way to determine the crack orientation.

## 4. Application to Field Data

**Figure 9:**
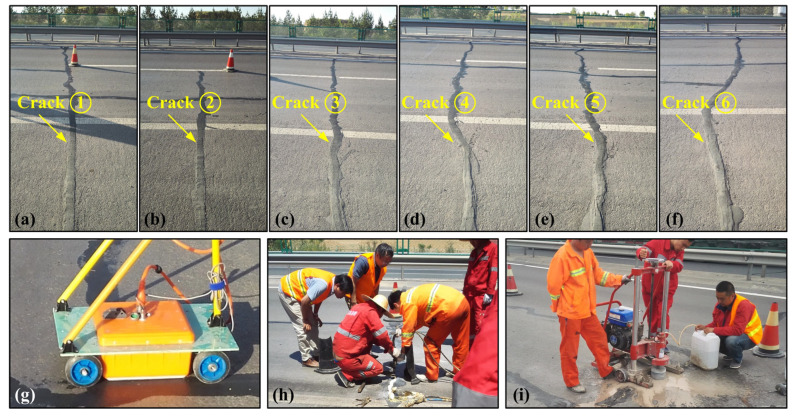
(**a**–**f**) Photo of six cracks, numbered ① to ⑥, along the highway to be detected. Note that all of the six cracks have been sealed with asphalt before the GPR survey. (**g**) GPR ground-coupled antenna used for the field data collection. (**h**) Non-excavation grouting site for repairing reflective cracks after GPR survey. (**i**) Core-drilling equipment.

The highway to be detected in this study consists of three layers: a 15 cm asphalt surface layer, a 36 cm semi-rigid base layer, and a fill subgrade. Figure 9a–f displays six visible cracks on the road surface, which have been sealed with asphalt by the maintenance organization before the GPR survey, a site where non-excavation grouting is used for crack repair (Figure 9h), and a location for core-drilling activities (Figure 9i). To collect the field GPR data, we employed the LTD-2600 system equipped with a ground-coupled antenna (Figure 9g) operating at 400 MHz, which is an intelligent GPR system newly developed by the China Academy of Radio Propagation in Qingdao, China. The system is built on an Android-based radar main unit and is compatible with a range of high-repetition-rate antennas. It includes robust data acquisition software that is suitable for various engineering inspection and geological exploration applications. The shielded antenna is mounted on a handcart (Figure 9g), allowing for smooth movement while maintaining a consistent position. This setup would ensure a stable antenna-to-ground distance of approximately 1 cm, preventing changes caused by surface irregularities. To enhance data collection further, we added a photoelectric encoder to the handcart, enabling us to acquire GPR data at equal distance intervals. Prior to data acquisition, we calibrated the encoder. Additionally, the trace spacing, acquisition time window, and time sampling interval were set to 3 cm, 40 ns, and 0.0781 ns, respectively.

**Figure 10:**
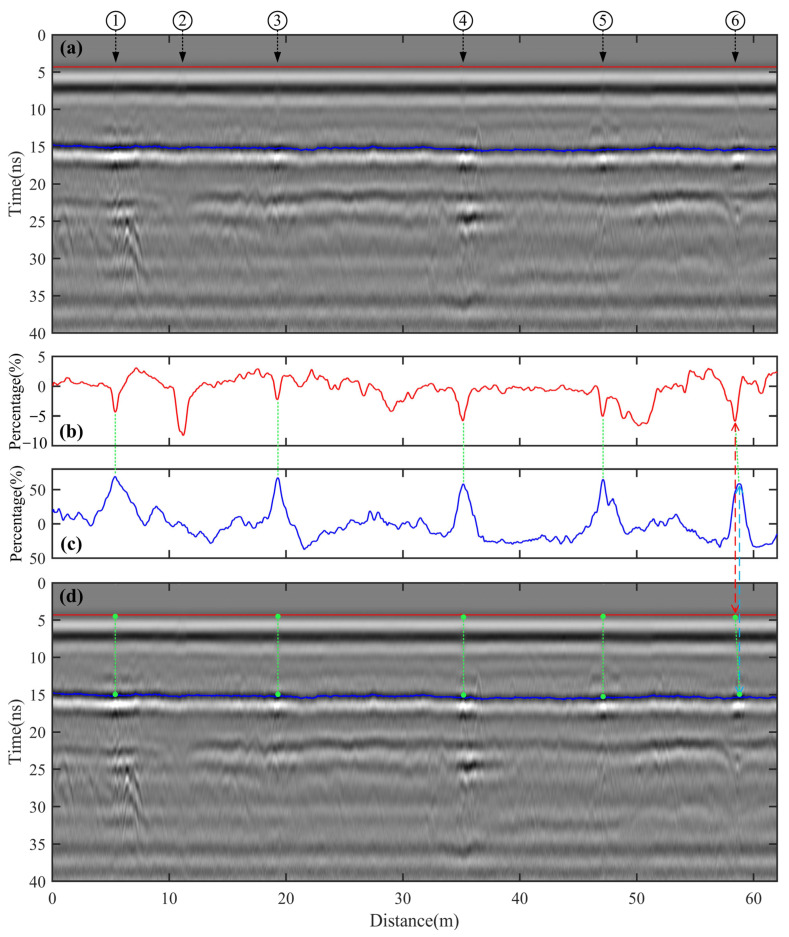
(**a**) The processed GPR profile using 400 MHz Ground-coupled Antenna. ①–⑥ represent the locations of the six cracks shown in Figure 9a and Figure 9f, Respectively. (**b**,**c**) The amplitude percentage change curves of the red and blue lines in (**a**). (**d**) The processed GPR profile using 400 MHz ground-coupled antenna with the locations of crack tops and bottoms plotted as green dots. In panels (**a**,**b**), the red and blue curves denote the first maximum trough amplitude of ETS and the maximum trough amplitude of the reflected wave generated at the interface between the semi-rigid base layer and subgrade. ①–⑥ represent the locations of the six cracks shown in Figure 9a and Figure 9f, respectively. The brown arrows point to the vertex of the “∨” and the crack top, while the cyan arrows point to the vertex of the “∧” and the crack bottom.

**Figure 11:**
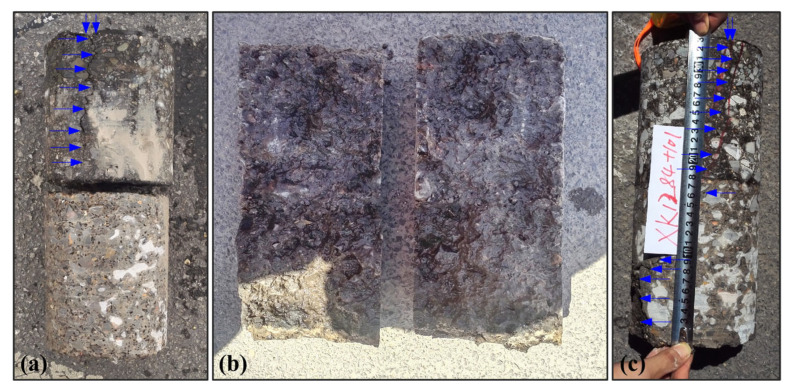
Core samples at the locations of cracks ② (**a**), ④ (**b**), and ⑥ (**c**) within the detected highway. The results reveal that cracks ②, ④ and ⑥ are a fatigue crack, reflective crack, and inclined reflective crack, respectively. The blue arrow indicates the location of the crack.

The radar survey line spans a total length of 62 m. After a regular preprocessing operation on the raw GPR data, including amplitude recovery and band-pass filtering, the processed GPR profile is shown in Figure 10a. Using the peak value method, the first maximum trough amplitude of the ETS and the maximum trough amplitude of the reflected wave at the interface between the semi-rigid base layer and the subgrade were tracked, as indicated by the red and blue solid lines, respectively. The amplitude percentage change curves corresponding to these lines are shown in Figure 10b and Figure 11c. These curves are based on the mode, which is the most frequently occurring value among the amplitude values.

In the GPR profile shown in Figure 10a, the diffracted waves of the crack tops are unclear, especially with the significant interference from the ETS, making it difficult to accurately identify the crack tops. However, the mixing and superposition of the diffracted waves from the crack tops with the ETS will inevitably cause changes in the first maximum trough amplitude of the ETS. In Figure 10b, the amplitude percentage change curve of the ETS shows a marked and sharp drop, forming a pronounced ‘∨’ shape at the top of the crack. This visualization provides a clearer and more intuitive representation of the crack locations compared to Figure 10a.

Additionally, the amplitude percentage change can be used to quantitatively estimate the crack width. In Figure 10b, the amplitude percentage changes for the six cracks are 4.38%, 8.25%, 2.26%, 5.82%, 5.07%, and 5.87%, respectively. Using these percentages and applying the formula Equation (4), the estimated crack widths are 6.28 mm, 11.32 mm, 3.32 mm, 8.21 mm, 7.21 mm, and 8.28 mm, respectively. Among these, the widths for crack tops are generally consistent with the on-site verification results, with errors within ±2 mm. This could be due to significant differences between the mean amplitudes on either side of these cracks and the reference baseline (mode of amplitude values).

In Figure 10c, the amplitude percentage change for crack ② is notably lower than that of the other five cracks. Based on numerical simulation results, it can be inferred that crack ② is a fatigue crack, with its bottom located at the interface between the asphalt surface and the semi-rigid base. In contrast, the other five cracks are reflective cracks, with their bottom located at the interface between the semi-rigid base and the fill subgrade. However, contrary to the numerical simulation results, Figure 10c shows that the amplitude percentage changes for the five reflective cracks are higher and more pronounced. This discrepancy might be due to the fact that after the cracks penetrate the asphalt surface layer and the semi-rigid base, water infiltrates along the cracks into the subgrade. Under repeated vehicle loading, this could cause a pumping effect, leading to water erosion around the bottom of the cracks and expanding their spatial extent. The larger the spatial extent of the crack bottom, the higher the peak and wider the base of the ‘∧’-shaped amplitude percentage change. This will make the difference in reflected wave amplitudes between reflective cracks and fatigue cracks at the interface between the semi-rigid base and the fill subgrade more pronounced, facilitating the identification of crack types.

Additionally, the crack top in Figure 10b appears as a sharp ‘∨’ shape, whereas the crack bottom in Figure 10c is represented as a sharp ‘∧’ shape. These shapes not only differ in form but also in relative vertex positions. By checking whether the vertices of these shapes align vertically, we can determine if the crack has experienced tilting. Thus, the line connecting the vertices of the ‘∨’ and ’∧’ shapes indicates the crack orientation. Core drilling can obtain accurate crack detection results at fixed points at the cost of causing damage to highway structure (Figure 9i). To verify the diagnostic results of the GPR data, three core samples were drilled from cracks ②, ④, and ⑥, and Figure 11 shows corresponding core samples. Note that core samples at cracks ② (Figure 11a) and ④ (Figure 11b) were both extracted before grouting, while core sample at cracks ⑥ (Figure 11c) was extracted after grouting. We can observe from Figure 11a that a clear crack (indicated by the blue arrows) extends from the road surface to the bottom of the asphalt layer (not reaching the top of the semi-grid base layer). Therefore, crack ② should be a fatigue crack, which fits our speculation. Figure 11b shows that crack ④ should be a reflective crack because the core sample has been split into two separate parts by the crack, and this result again validates our detection result on crack ④. In Figure 11c, we can find that the crack has spread across the whole asphalt and semi-grid base layer from the road surface, which is indicated by the blue arrows. Moreover, the core sample results show that the crack may have a certain degree of tilting. Crack ⑥ is then identified as an inclined reflective crack, which is consistent with our result. Overall, the crack detection results based on GPR data are in good agreement with the corresponding core sampling results, demonstrating the validity and effectiveness of our proposed method.

## 5. Discussion

Cracks are not only a common and typical defect in roads but also give rise to other issues such as mud pumping, water-rich subgrade, and void formation. Common mobile detection technologies, such as lasers, infrared thermography, remote sensing, and cameras, can accurately measure the geometric attributes and distribution of cracks on the road surface. However, their detection depth is limited, especially when the top of the crack is covered with asphalt binder, making it impossible to detect the internal condition of cracks within the road structure layers. The width of cracks is usually only a few millimeters, which is far less than the lateral resolution of ground-penetrating radar (GPR). Therefore, the application of GPR in road crack detection has been limited to qualitative interpretation. This paper provides a more comprehensive and accurate identification of crack characteristics, including the top, bottom, width, and orientation, by analyzing the amplitude of the ETS and the reflected waves from the interface between the semi-rigid base and the subgrade.

The ETS, which is the first-arrival wave collected by ground-coupled radar, often obscures the diffracted waves emanating from the tops of cracks, complicating the precise localization of these crack tops within the GPR profile. However, the existence of a crack has a specific effect on the ETS. The amplitude of the ETS locally increases at the crack location [17]. This finding aligns with the conclusion that a lower dielectric constant of the surface medium leads to a higher amplitude of the corresponding ETS [25]. In contrast to the AEA statistical method, the ETS exhibits a pronounced ‘∨’-shaped dip in its first maximum trough amplitude at the crack top. This ‘∨’-shaped dip serves as a clear and intuitive marker for the crack top, simplifying its identification [30]. In this paper, our research has further revealed a positive correlation between crack width and the amplitude strength of the ETS at the ‘∨’ vertex. This correlation allows for the quantitative estimation of crack width. At the interface between the semi-rigid base and the subgrade, the crack’s bottom is marked by a sharp ‘∧’-shaped dip in the reflected wave. For cracks of identical width, the percentage change in amplitude caused by reflective cracks is approximately three times greater than that caused by fatigue cracks, providing a more accurate quantitative basis for identifying the crack bottom and diagnosing crack types. By analyzing the line connecting the vertices of the ‘∨’ and ‘∧’ shapes (representing the crack’s top and bottom, respectively), the crack’s orientation can be determined easily.

To accurately and reliably evaluate the attribute characteristics of road cracks with GPR, attention should be paid to the following points. Firstly, it is essential to couple the shielded antenna to the ground, with the antenna positioned as close to the road surface as possible, to maximize the penetration of electromagnetic waves into the road structure. Secondly, the offset distance of the antenna should be minimized to ensure that airwaves and ground waves can superimpose, jointly comprising the early signals of the GPR. Additionally, the distance between the antenna and the road surface should be kept as constant as possible to stabilize the waveform and amplitude of the ETS, accurately reflecting changes in the electromagnetic parameters of the shallow subsurface medium at the road surface. Lastly, the spacing between survey lines should be minimized to enable more scan lines to cover the cracks.

Accurate detection and characterization of cracks using GPR have practical implications for road maintenance. GPR provides detailed information on crack attributes such as top, bottom, width, and orientation, enabling the development of precise and minimally invasive repair measures. For fatigue cracks in the asphalt surface layer, repair can be achieved through slot-cutting and filling with asphalt sealant, which prevents further deterioration of the cracks and asphalt aging. In the case of reflective cracks, a trenchless grouting method can be used to fill the cracks with high-strength polymers that exhibit elasticity, viscosity, and strength. This method connects the fractured semi-rigid base layer with the asphalt surface, thereby preventing water infiltration into the subgrade. When reflective cracks are inclined, the position of the grouting holes can be adjusted to align with the crack bottom, enabling the high-strength polymer to fill the cracks from bottom to top. Addressing cracks promptly and accurately helps prevent moisture infiltration into the subgrade, extending the road’s service life.

Given that roads within a certain length share the same material type, paving time, environmental impacts, and vehicular load effects, this study assumes that the ETS and reflected wave amplitudes obtained from GPR for normal road structural layers (i.e., roads without cracks) are stable. These parameters are utilized as intermediate reference values to calculate the percentage change in amplitude, which is then employed for the quantitative assessment of crack top width and crack type (namely, fatigue cracks and reflective cracks). However, this method may become inaccurate when there are significant local abrupt changes in the medium properties of the road structural layer. Additionally, it should be noted that the crack widths calculated in this study are limited to the widths of the crack tops and do not represent the full width of the entire crack. Furthermore, the crack models employed in the numerical simulation exhibit regular geometric shapes, with the tops and bottoms of the cracks situated at the interfaces of the road’s structural layers. This methodology does not fully or accurately capture the actual state of road cracks. When the crack is filled with a material other than air, it will also affect the amplitude of the diffraction waves at the top and bottom of the crack, thus impacting the sensitivity of this method.

The results of this study provide a basis for detecting key parameters such as the top, bottom, width, and orientation of cracks. However, follow-up work will primarily focus on two aspects to expand and refine this research. Firstly, based on the discrete random distribution characteristics of road materials, a multiphase discrete random medium model that more closely aligns with the real conditions of road materials will be established to numerically simulate the wave field response characteristics and patterns of cracks. Additionally, we will investigate how different environmental conditions, such as temperature and humidity, affect GPR signals in order to improve the accuracy of crack interpretation.

## 6. Conclusions

The study demonstrates the effectiveness of GPR in identifying various crack characteristics, such as top, bottom, orientation, and width. This is achieved through the analysis of the ETS amplitude and the reflected wave from the interface between the semi-rigid base and the subgrade. The analysis yields five key findings, which can be summarized as follows:

- The ETS shows a ‘∨’-shaped dip in its first trough at the crack top, serving as a clear marker for easy identification.
- A positive correlation exists between crack width and ETS amplitude at the ‘∨’ vertex, enabling quantitative crack width estimation.
- At the semi-rigid base-subgrade interface, a ‘∧’-shaped dip in the reflected wave signals a crack bottom. Reflective cracks of the same width cause an amplitude change three times that of fatigue cracks, offering a quantitative basis for identification and diagnosis.
- By analyzing the line connecting the ‘∨’ and ‘∧’ vertices (crack’s top and bottom), the orientation can be easily determined.
- The proposed GPR method accurately detects crack attributes, enabling precise and minimally invasive repair strategies for fatigue and reflective cracks, which prevents further deterioration, delays asphalt aging, prevents water infiltration, and extends road service life.

## Data Availability

The data presented in this study are available on request from the corresponding author. The data are not publicly available due to some special reasons.
